# Pathogenic *Yersinia* Promotes Its Survival by Creating an Acidic Fluid-Accessible Compartment on the Macrophage Surface

**DOI:** 10.1371/journal.pone.0133298

**Published:** 2015-08-14

**Authors:** Wael Bahnan, Douglas R. Boettner, Linda Westermark, Maria Fällman, Kurt Schesser

**Affiliations:** 1 Department of Microbiology and Immunology, University of Miami Miller School of Medicine, Miami, Florida, United States of America; 2 Department of Molecular and Cellular Pharmacology, University of Miami Miller School of Medicine, Miami, Florida, United States of America; 3 Department of Molecular Biology, Umeå University, Umeå, Sweden; University of Louisville, UNITED STATES

## Abstract

Microbial pathogens and host immune cells each initiate events following their interaction in an attempt to drive the outcome to their respective advantage. Here we show that the bacterial pathogen *Yersinia pseudotuberculosis* sustains itself on the surface of a macrophage by forming acidic fluid-accessible compartments that are partially bounded by the host cell plasma membrane. These *Yersinia*-containing acidic compartments (YACs) are bereft of the early endosomal marker EEA1 and the lysosomal antigen LAMP1 and readily form on primary macrophages as well as macrophage-like cell lines. YAC formation requires the presence of the *Yersinia* virulence plasmid which encodes a type III secretion system. Unexpectedly, we found that the initial formation of YACs did not require translocation of the type III effectors into the host cell cytosol; however, the duration of YACs was markedly greater in infections using translocation-competent *Y*. *pseudotuberculosis* strains as well as strains expressing the effector YopJ. Furthermore, it was in this translocation- and YopJ-dependent phase of infection that the acidic environment was critical for *Y*. *pseudotuberculosis* survival during its interaction with macrophages. Our findings indicate that during its extracellular phase of infection *Y*. *pseudotuberculosis* initiates and then, by a separate mechanism, stabilizes the formation of a highly intricate structure on the surface of the macrophage that is disengaged from the endocytic pathway.

## Introduction

Phagocytic cells such as macrophages fulfill a variety of functions including direct pathogen killing, secreting cytokines, and displaying pathogen-derived antigens on their surface. Pathogens can disrupt, or in some cases subvert, these macrophage functions to either promote their immediate survival and/or to derail the system-level immune response. A clear instance of pathogen manipulation of host cell function are the type III secretion systems possessed by several of the most prevalent Gram-negative bacterial animal and plant pathogens. These bacterial organelles deliver proteins (termed ‘effectors’) into the eukaryotic cell cytosol [1]. Individual pathogens secrete a largely unique set of effectors that drive the interaction either to the advantage of the microbe or, in certain cases, the host, depending on the context of the infection. For example, among human pathogens, *Shigella* and *Salmonella* employ their type III systems to invade and replicate within host cells, whereas the extracellular pathogens *Yersinia* and *E*. *coli* employ theirs to counteract phagocytosis [2]. Despite this wide variety in their usage, type III secretion systems are used by bacteria that are in close physical contact with eukaryotic cells.

There are three species of the Gram-negative *Yersinia* that are human pathogens. *Y*. *pseudotuberculosis* and *Y*. *enterocolitica* are food-borne pathogens that infect the gastrointestinal tract whereas *Y*. *pestis*, the causative agent of bubonic and pneumonic plague, is a vector-borne pathogen that infects the blood and lymphatic systems. In order to gain access to deeper tissue, *Y*. *pseudotuberculosis* and *Y*. *enterocolitica* must first cross the cells lining the gastrointestinal tract: this aspect of the infection process is independent of their respective type III secretion systems. Recently it was shown that when cultured epithelial-like cells are infected with a *Y*. *pseudotuberculosis* derivative strain lacking the type III secretion system-encoding virulence plasmid, a ‘pre-vacuole’ compartment is created along the bacterial-host cell interface which is accessible to the extracellular environment [3]. The duration of this compartment is relatively brief (<10 mins) and following its closure the bacteria were phagocytosed and the nascent phagosome appeared to mature normally.

Here we examined the interaction between virulent *Y*. *pseudotuberculosis* (i.e., possessing its type III secretion system-encoding virulence plasmid [4]) with cells of the innate immune system. Our results show that *Y*. *pseudotuberculosis* uses virulence plasmid-encoded factors to drive the formation of stable structures on the surface of macrophages that superficially resemble the previously described pre-vacuoles. However, in several key respects these structures differ from those that form between plasmid-cured *Yersinia* and epithelial cells [3]; most critically, the intimate interaction between virulent *Y*. *pseudotuberculosis* and macrophages does not, in the majority of cases, lead to the formation of a phagosome. Our results indicate that *Y*. *pseudotuberculosis* has evolved to promote its survival by undermining a fundamental macrophage function.

## Materials and Methods

### Cell culture and reagents

The murine RAW 264.7 macrophage-like cell line was cultured in DMEM supplemented with 10% fetal bovine serum (FBS). The day before infection, 10^5^ RAW cells were seeded onto 12mm glass coverslips for microscopy or directly into 24-well tissue culture dishes for CFU assays. Bone marrow-derived macrophages (BMMs) were prepared as previously described [5]. Peritoneal exudate macrophages (PEMs) were isolated from C57BL/B6 mice as described [6] and were seeded either on glass coverslips or into 24-well tissue culture dishes. Mice were treated humanely in strict accordance with federal and state government guidelines for the Care and Use of Laboratory Animals of the National Institutes of Health and their use was approved for this entire study by the University of Miami institutional animal care and use committee (protocol number 11–186). Cells were allowed to adhere to the glass or plastic and the experiments were done either the same day or the next day. In Dynasore, ammonium chloride or Tris buffering experiments, the macrophages were pre-treated with 80μM Dynasore, 30mM NH_4_Cl or 30mM Tris pH 7.45 for 60 minutes of infection and were kept in the same media the duration of the infection.

### Bacterial culture and infection conditions

*Yersinia pseudotuberculosis* strains YPIII/pYV (pIB102; [7]), its virulence plasmid-deleted derivative YPIII/ΔpYV, or YPIII/pYV/*ΔyopB* (pIB604; [8]) and YPIII/pYV/*ΔyopJ* (pIB232; [9]) were used in this study. The pGFP plasmid (Clonetech), which constitutively expressed the GFP protein was transformed into *Y*. *pseudotuberculosis* strains to enhance their visualization in microscopy-based experiments. The *yopJ* mutation was created in the YPIII/pYV/*ΔyopB* strain using the lambda red recombination method [10], giving rise to the YPIII/pYV/*ΔyopBΔyopJ* double mutant. For the ELK phosphorylation experiments, the plasmid expressing *yopE*_*1–120*_ *-ELK* [11] was transformed into the YPIII/pYV and YPIII/ΔpYV strains. For infections, the bacteria were grown overnight in DMEM+10%FBS at 27°C in a shaking incubator. The overnight culture was diluted 1:40 in DMEM+10% FBS and grown for 2 hours at 27°C before being switched into a 37°C shaking incubator for one hour to induce the expression of type III-encoding genes. For the translocation assay, cells were infected for 2 hours and then washed, lysed and the resulting lysates were analyzed using an anti-phospho-Elk(Ser383) and Elk antibodies (Cell Signaling) to detect phosphorylated and total Elk, respectively, by western blotting. To determine the viability of bacteria associated with macrophages (i.e., CFU assay), the macrophages were infected for the indicated time, washed 3–4 times with PBS and lysed with sterile distilled water. The lysates were vortexed and serial dilutions were plated and the resulting CFU enumerated two days later. In certain experiments gentamicin was added at a final concentration of 2μg/ml for 30 minutes before cell lysis.

### Labeling conditions and microscopy

pHrodo (Red Dextran 10,000MW; Invitrogen) is an acid sensitive fluorescent dye which has a linear increase in fluorescence under acidifying conditions and thus can be used as a pH indicator for cellular compartments. Macrophages were washed with phosphate buffered saline (PBS) and then labeled with pHrodo (diluted in PBS supplemented with 10% FBS) at a final concentration of 10μg/ml for 40 minutes. For the simultaneous labeling and infection experiments, bacteria were washed with PBS and reconstituted in PBS+10% FBS before being added to the macrophages which were treated as before. Following labeling, macrophages were fixed with 3.7% paraformaldehyde for 12 minutes before being washed and mounted onto pre-cleaned glass slides. DAPI was included in the Pro-Long Gold mounting medium (Invitrogen). Microscopy was carried out on an Olympus fluorescence BX61 upright microscope equipped with Nomarski differential interference contrast (DIC) optics, a Uplan S Apo 100x objective (NA 1.4), a Roper CoolSnap HQ camera, and Sutter Lambda 10–2 excitation and emission filter wheels, and a 175 watt Xenon remote source with liquid light guide. Image capture was automated using Intelligent Imaging Innovations Slidebook 4.01 for the Mac. For all the cells analyzed, a series of optical Z-sections (0.35 μm) were captured. Prior to analysis individual stacks were deconvolved using the nearest neighbor algorithm. Representative projected images were chosen to be included in the figures. For quantification of fluorescent signal, ImageJ software was used to quantify the fluoresce signal per infected cell or per bacterium. The fluorescence signal was divided by the area of the cell or bacterium to generate a signal/area ratio that was termed ‘fluorescence intensity in arbitrary units’. The percentage of bacteria co-localized with pHrodo* (i.e., red-emitting pHrodo using identical brightness/contrast settings for all slides) was generated by counting >40 cells in at least two independent experiments, and the number of (macrophage-associated) bacteria associated with pHrodo* signal were divided by the total number of bacteria (latter visualized by DAPI). To generate the ratio of intracellular bacteria, each Z-stack image of the cells was examined for the localization of the macrophage associated bacteria. Bacteria which were on the extracellular side of the membrane were termed extracellular, bacteria which were clearly internalized and had dislodged from the inner face of the membrane were counted as intracellular. For CD11b, EEA1 or LAMP1 straining, the infected macrophages were fixed with 3.7% paraformaldehyde, permeabilized with PBS+0.1% Triton X-100, and blocked with PBS + 3% Bovine serum albumin. The cells were then stained with antibodies specific for CD11b (eBioscience 14-0112-82), EEA1 (Abcam 2900) or LAMP1 (Abcam 24170) for 60 minutes at room temperature. The cells were then stained with secondary antibodies conjugated to AlexaFluor 555 for one hour at room temperature before being washed and mounted on slides. For laser confocal scanning microscopy, a PlanApo N 60x/1.42 NA oil-immersion objective was used on an Olympus FluoView FV1000 confocal microscope (Olympus, Tokyo, Japan). Confocal image stacks (12–20 images per stack) were acquired at 800 × 800 pixel resolution and a step size of .50 μm. Images were processed using ImageJ software (NIH, Bethesda, MD) and Photoshop (Adobe Systems, CA) to demonstrate z-slices. Live cell microscopy was performed as described [9]. Briefly, infected BMMs were imaged by differential interference contrast (DIC) using a Nikon Eclipse Ti-E microscope using an Andor iXon + EMCCD camera. Images were captured every 5 seconds for 30 minutes and were collected into a video with a display rate of 9 frames/second. Infected cells were prepared for scanning electron microscopy (SEM) by first fixing in 2.5% glutaraldehyde (in PBS) prior to casting onto poly-lysine coated glass slides that were then dehydrated in a series of graded ethanol. Dehydrated samples underwent critical point drying and followed by a 5-nm coating of iridium. SEM was performed at the Umeå Core Facility for Electron Microscopy (UCEM) with a Carl Zeiss Merlin Field Emission Scanning Electron Microscope (FESEM) using in-lens secondary electron detector at beam accelerating voltage of 4 kV and probed with a 90 pA current. Data collection was with an Oxford Instruments X-MAX 80 mm2 X-ray detector and recorded using SmartSEM V.5.07 software.

## Results

### Pathogenic *Yersinia* establishes an extracellular niche on the macrophage surface

**Fig 1:**
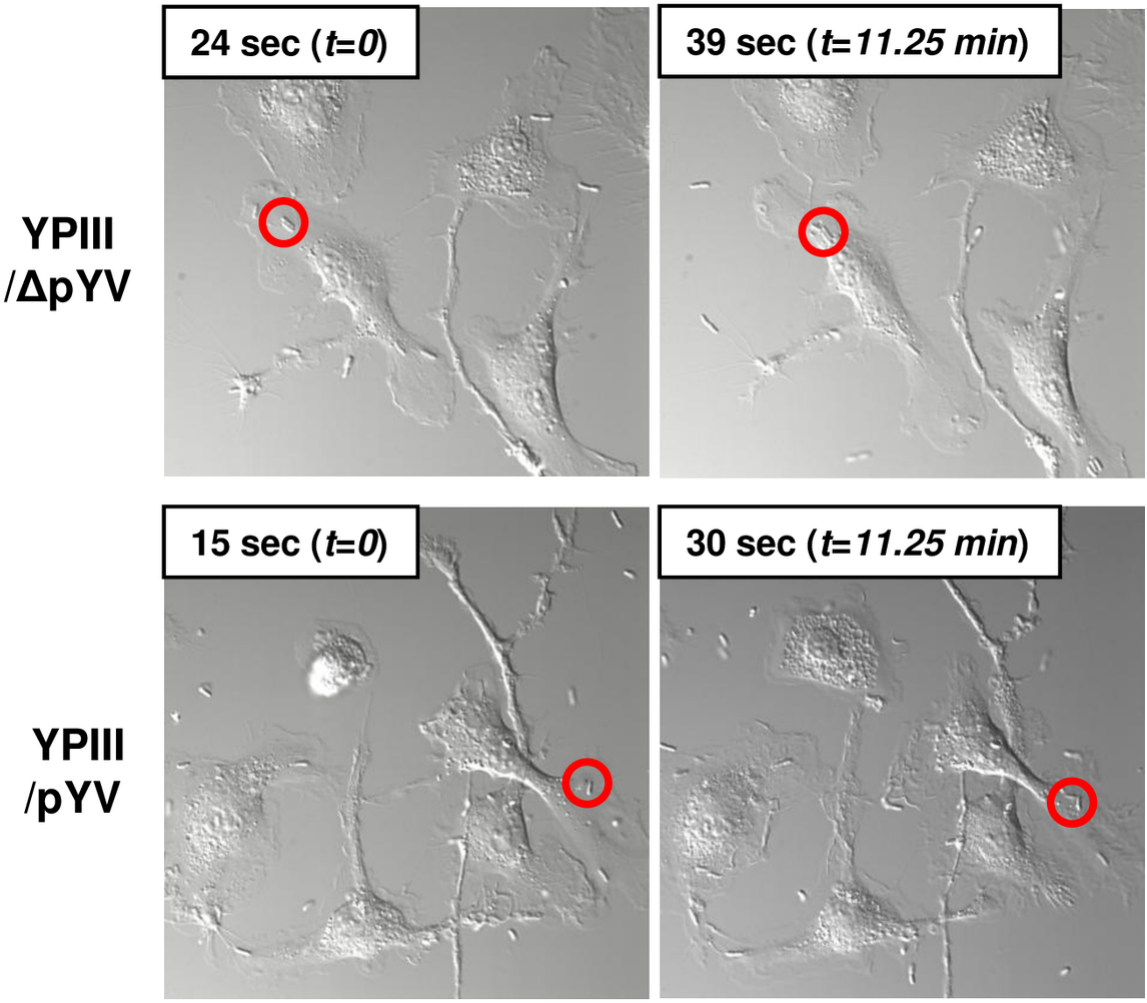
Virulent *Yersinia* sustains itself on the macrophage surface. Bone marrow-derived macrophages were infected with *Y*. *pseudotuberculosis* YPIII strains either lacking or harboring the pYV virulence plasmid (designated as YPIII/ΔpYV and YPIII/pYV, respectively). Infections were monitored by time-lapse microscopy for 30 mins and shown are two grabs from each infection (video files at http://youtu.be/YcA-eXEdfUA and http://youtu.be/6PlOS_hc2ww). (*top*) Highlighted in the left panel is a YPIII/ΔpYV bacterium at movie time 24 secs that becomes completely phagocytosed within 15 secs (in real time equivalent to 11.25 minutes). (*bottom*) Highlighted is a YPIII/pYV bacterium that remains on the surface of the macrophage for a comparable amount of time.

Using time-lapse microscopy we analyzed the interaction between *Y*. *pseudotuberculosis* and macrophages. The *Y*. *pseudotuberculosis* strain YPIII lacking the pYV virulence plasmid (designated as YPIII/ΔpYV) was swiftly taken up by bone marrow-derived macrophages (Fig 1 and video files http://youtu.be/YcA-eXEdfUA). In contrast, following its contact with macrophages, *Y*. *pseudotuberculosis* bacteria harboring the pYV virulence plasmid (YPIII/pYV) resided on the cell surface in several instances for over 10 minutes without being phagocytosed (Fig 1 and video files http://youtu.be/6PlOS_hc2ww).

**Fig 2:**
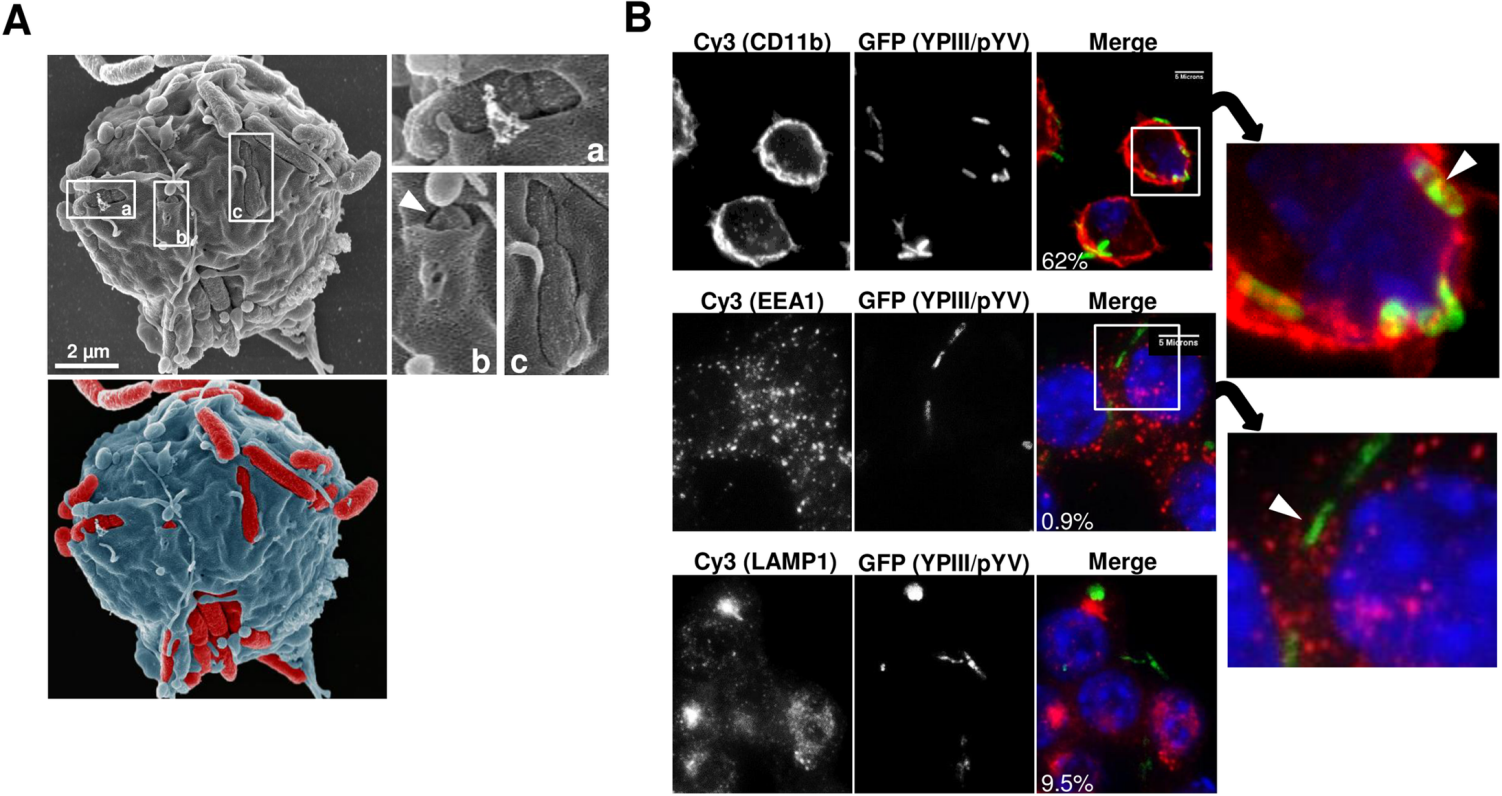
Virulent *Yersinia* becomes partially embedded within the macrophage plasma membrane. (A) RAW macrophages were infected with YPIII/pYV for 100 minutes and analyzed by scanning electron microscopy (SEM). Shown is a representative image depicting a single macrophage infected with several YPIII/pYV bacteria. Shown in the enlargements are bacteria partially enveloped within host membrane. The bacterium in panel b is indicated by an arrow. Shown below is a false coloring rendering highlighting the demarcation of the host cell (blue) and YPIII/pYV bacteria (red). (B) Macrophages were infected with YPIII/pYV for 100 minutes and then stained for the plasma membrane marker (CD11b), the early endosomal marker (EEA1), and a lysosomal marker (LAMP1) and analyzed by fluorescent microscopy. The arrow in the enlarged image of the CD11b-stained cells indicates a bacterium closely associated with host cell membrane; 62% of macrophage-associated bacteria (N = 56) were similarly observed. Shown in the right panels of the EEA1- and LAMP1-stained cells is the percentage of bacteria-containing endosomes that stained for either EEA1 or LAMP1 (N = 111 and 52, respectively). The arrow in the enlarged image indicates a bacterium distinct from an EEA1-staining compartment. The data shown is representative of 3 independent experiments with similar results using both RAW macrophages and murine peritoneal macrophages.

Scanning electron microscopy (SEM) was used to visualize the configuration of YPIII/pYV bacteria on the macrophage surface during their prolonged interaction. Macrophage-associated YPIII/pYV bacteria displayed various levels of association with host plasma membrane: ranging from partial embedment (Fig 2A; *enlargements a and c*) to being nearly fully enclosed (*see arrow*, *enlargement b*). Using similar conditions we were unable to observe YPIII/ΔpYV bacteria on macrophage surfaces presumably because of their rapid internalization. Although as expected macrophage-associated YPIII/pYV bacteria co-localized with the membrane marker CD11b (Fig 2B; *note arrow in enlarged panel*), however, these YPIII/pYV bacteria co-localize with neither EEA1, which normally accumulate in early endosomes, nor LAMP1, which accumulates on the plasma membrane following membrane injury [12]. Collectively these findings indicate that despite their sustained residency on the surface of macrophages and becoming partially enclosed within host cell membrane, YPIII/pYV bacteria are able to prevent the macrophage from executing phagocytosis.

### *Yersinia*-containing acidified compartments (YACs) are created in infected macrophages

**Fig 3:**
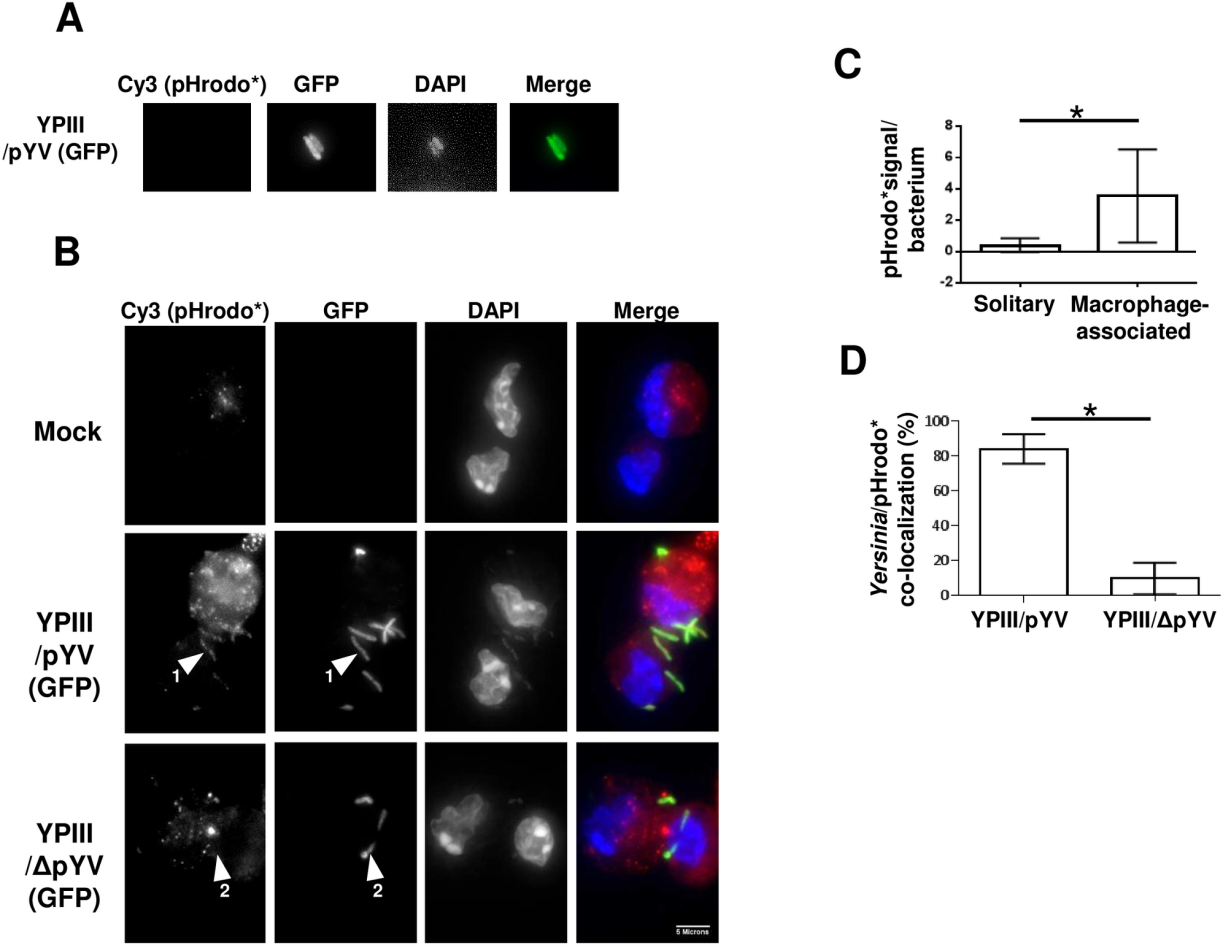
*Yersinia*-containing acidified compartments (YACs) form on the macrophage surface. (A) Solitary wild-type (pYV) *Y*. *pseudotuberculosis* bacteria from the same slide shown in (B) do not co-localize with red-fluorescing pHrodo (designated as pHrodo*). (B) Peritoneal macrophages were simultaneously labeled with pHrodo and infected with GFP-expressing wild-type (pYV) or plasmid-cured (pΔYV) *Y*. *pseudotuberculosis* for 40 minutes and then analyzed by fluorescent microscopy. The arrows in the middle row images indicates a YPIII/pYV bacterium that is intensely positive for pHrodo* in contrast to the pΔYV bacterium indicated with arrows in the bottom row. (C) The pHrodo* signal associated with >20 YPIII/pYV bacteria which were either solitary or cell-associated (shown in A and B, respectively) was quantified using Image J and normalized to the slide background. (D) The percentage of *Y*. *pseudotuberculosis* co-localized with acid-positive pHrodo* was generated by counting bacteria associated with >40 infected cells in multiple independent experiments. (* *P* < 0.05 using student *t*-test)

To examine the environment at the *Yersinia*-macrophage interface, macrophages were labeled with dextran-conjugated pH-sensitive rhodamine (pHrodo) either in the absence or presence of *Y*. *pseudotuberculosis*. Acidification of the pHrodo label leads to a linear increase in its fluorescence starting at a pH of 6.8 and reaching a maximum at pH 5 (red-emitting pHrodo will henceforth be referred to as ‘pHrodo*’). There is an extremely low level of pHrodo* signal associated with solitary YPIII/pYV bacteria (Fig 3A; *and see below*). In uninfected primary macrophages, pHrodo is taken up by cells through endocytosis, eventually becoming acidic in the late endosome and lysosomal compartments indicated by an intense red signal in the perinuclear region (Fig 3B; *top row*). In macrophages simultaneously labeled with pHrodo and infected with YPIII/pYV, in addition to the pattern of pHrodo* fluorescence observed in uninfected cells, a pHrodo*-based fluorescent coat formed around the majority of the bacteria along the periphery of the macrophage (84%) as indicated by a highly concentrated signal enveloping the bacterial cell (Fig 3B; *middle row*, *arrow 1*). The pHrodo* signal of macrophage-associated bacteria was several-fold higher than the barely detected pHrodo* signal associated with solitary bacteria (Fig 3C). The magnitude of the pHrodo*-based fluorescence of individual macrophages was directly proportional to the number of associated YPIII/pYV bacteria indicating that the pHrodo* signal is primarily due to infection (S1 Fig). In striking contrast, a significantly smaller fraction (9.5%) of macrophage-associated YPIII/ΔpYV bacteria created this acidic environment (Fig 3B; *bottom row*, *arrow 2*). In similar experiments using the murine macrophage-like cell line RAW 267.4, 90% percent of YPIII/pYV bacteria were in pHrodo* acidified compartments (henceforth designated as *Y* *ersinia*-containing acidic compartments, YACs), whereas only 10% of YPIII/ΔpYV bacteria were located in YACs (*P* <0.05, student t-test; *data not shown*). In contrast to the potent pHrodo* signal observed in infected macrophages, there was an extremely low pHrodo* signal in YPIII/pYV-infected epithelial cell lines (HeLa and COS) that was difficult to distinguish from the background (*data not shown*).

**Fig 4:**
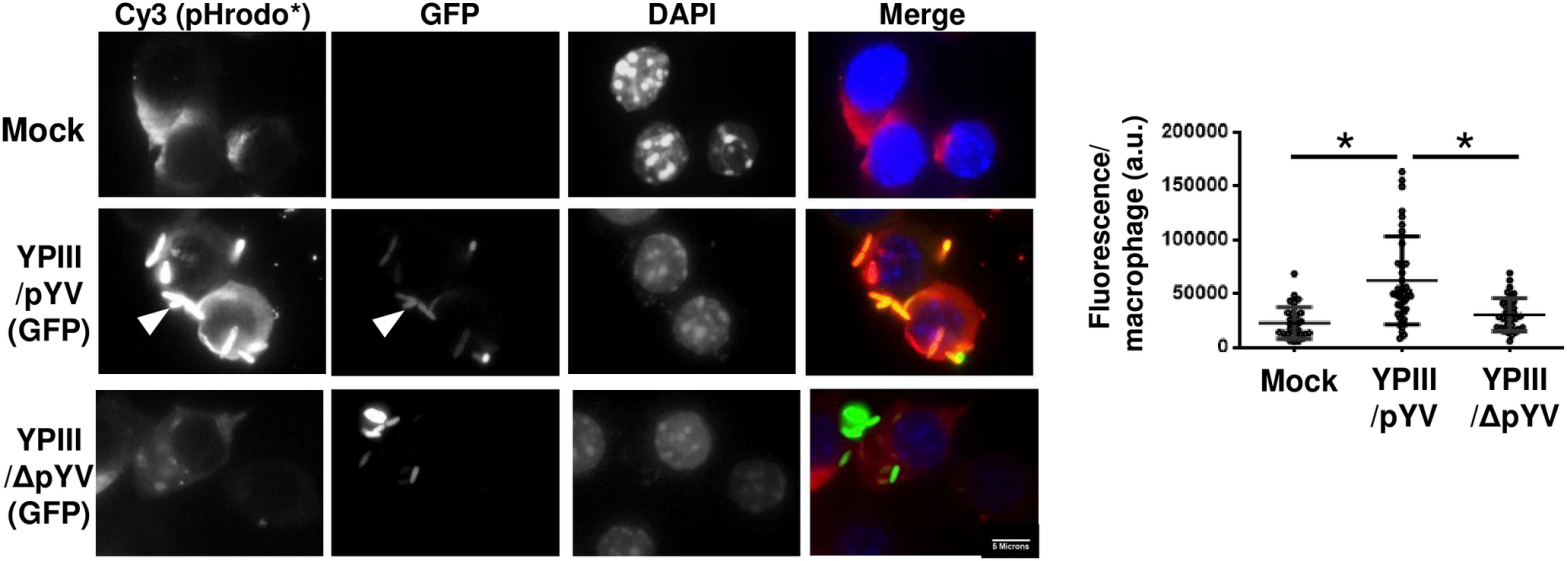
YACs are stably confined macrophage surface structures. RAW macrophages were infected for 60 minutes with GFP-expressing wild-type (pYV) or plasmid-cured (pΔYV) *Y*. *pseudotuberculosis*. pHrodo was then added to the infected cells for 40 minutes and were then analyzed as in Fig 3 and what is presented is representative of multiple experiments. The arrows in the middle row images indicates a YPIII/pYV bacterium that is intensely positive for pHrodo*. Plotted to the right is the corresponding fluorescence of individual macrophage-associated bacteria. (N >40 each condition; * *P* < 0.05 using student *t*-test)

**Fig 5:**
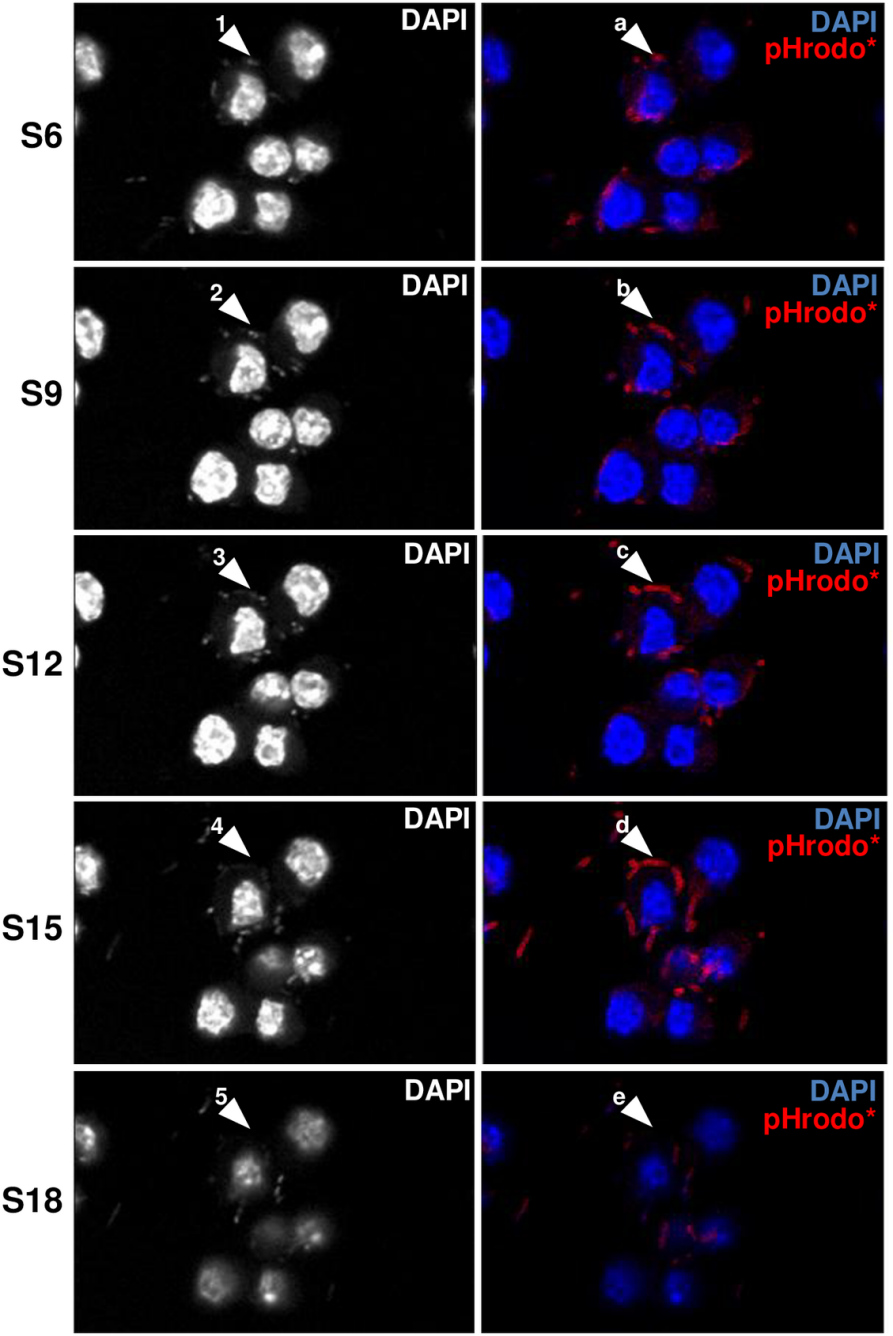
Surface-bound *Yersinia* is tightly enclosed in a low pH environment. RAW macrophages were infected with wild-type *Y*. *pseudotuberculosis* (pYV) for 40 minutes and then labeled with pHrodo as described in Fig 4 and then analyzed by laser scanning confocal microscopy. Shown are 5 representative planes of a series of 12 consecutive Z-stacks (S) in which each stack is 0.5μm from the subsequent slice. See main text for explanation of the indicated arrows.

To assess the stability of YACs, macrophages were infected with YPIII/pYV or YPIII/ΔpYV bacteria for 60 minutes and then followed by a 40-minute pHrodo labeling period. This infection/labeling regime resulted in highly pHrodo* fluorescent YPIII/pYV bacteria (Fig 4; *note arrow in middle row*). In fact the pHrodo* fluorescent coating observed around the surface-bound YPIII/pYV bacteria after this 100 minutes infection period (60 + 40) was much more pronounced compared to the 40 minute infection experiment (see Fig 3B) suggesting that YACs become increasingly more acidic during infection. Additionally, these data indicate that following their formation, YACs remain accessible to the extracellular milieu; this issue will be further addressed below. Laser scanning confocal microscopy was employed to precisely characterize the spatial relationship between the pHrodo* signal and YPIII/pYV bacteria (due to rapid bleaching of GFP, bacteria were visualized by DAPI staining). As highlighted in Fig 5 in which successive Z-stacks planes are examined, there is little bacterial-derived DAPI signal and pHrodo* signal in the first Z-stack examined (*arrows 1 and a*). In the succeeding Z-stacks, a distinct DAPI signals (*arrows 2 and 3*) are immediately adjacent to the pHrodo* signals (*arrows b and c*) that is followed by a pHrodo*-only signal (*arrow d*). In the final Z-stack examined, there is neither DAPI nor pHrodo* signal (*arrows 5 and e*, respectively). These data indicate that an acidic environment is closely associated with YPIII/pYV bacteria and when considered with the imaging data shown in Fig 2A, suggest that this pathogen forms an acidic environment between it and the host plasma membrane.

To determine whether the formation of these acidified compartments depended on host cell trafficking, cells were treated with an inhibitor of dynamin (Dynasore) to impair endosomal transport. In addition to preventing post-endocytic scission, the inhibition of dynamin causes a defect in membrane fission resulting in impaired acidification of late endosomes and lysosomes [13]. Treating macrophages with the dynamin inhibitor for 60 minutes completely eliminated pHrodo uptake through the normal endocytic pathway; however, such cells, when infected with YPIII/pYV bacteria, still formed highly fluorescent YACs with similar kinetics as untreated infected macrophages (*data not shown*). Collectively these data indicate that YACs are not coupled to the macrophage endocytic trafficking pathway.

### YAC formation does not require delivery of the type III effectors into the host cell cytosol

**Fig 6:**
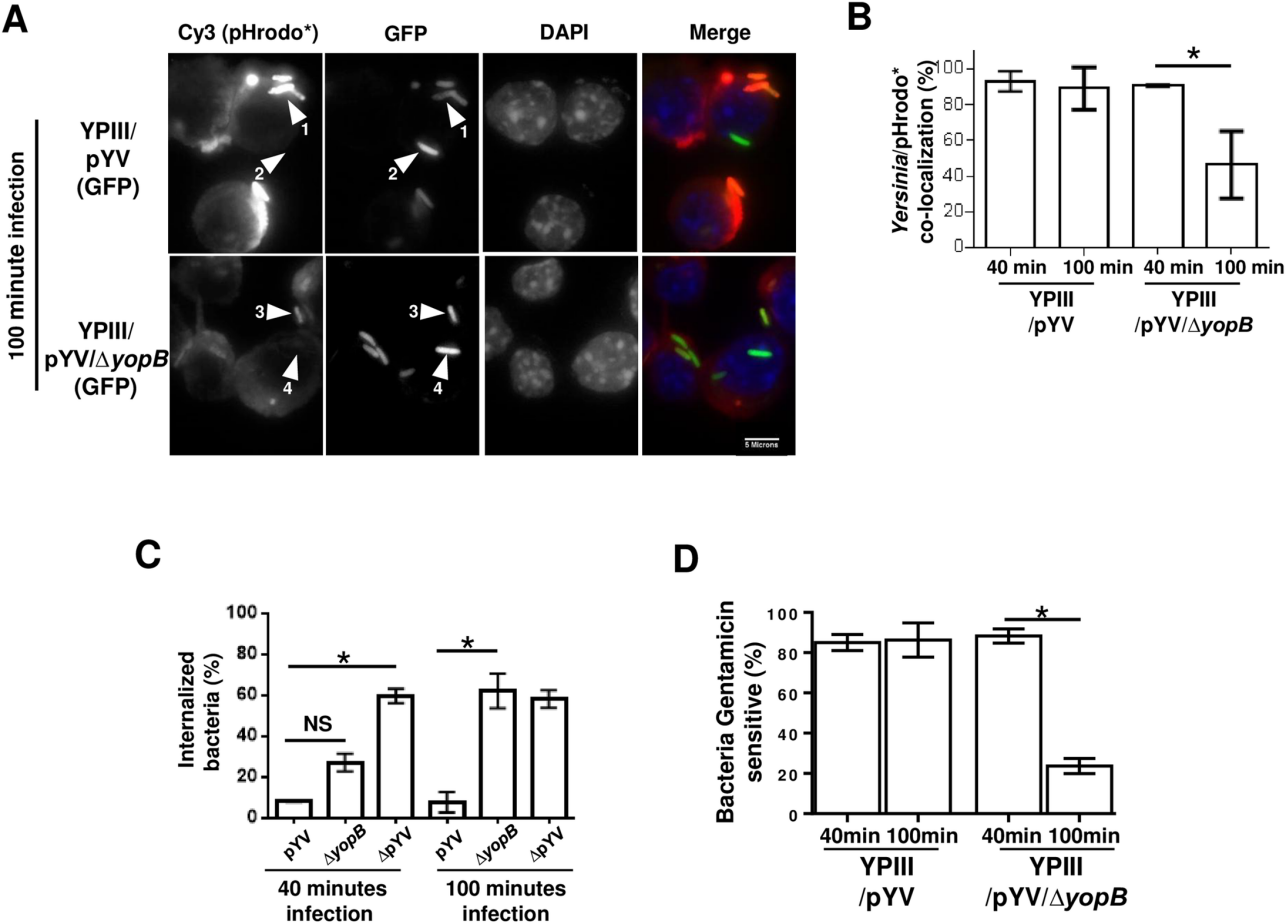
Secretion of type III effectors is sufficient to create but not maintain YACs. (A) Peritoneal macrophages were infected with GFP-expressing wild-type (YPIII/pYV) or a type III translocation mutant (YPIII/pYV/Δ*yopB*) strain for 60 minutes prior to the addition of pHrodo and incubated for an additional 40 minutes of infection (= 100 mins total infection). See main text for explanation of the indicated arrows. (B) The percentage of the bacteria co-localizing with the pHrodo signal from 40 or 100 mins of infection are shown from counting >30 macrophages from a single representative experiment that was repeated multiple times. (C) Peritoneal macrophages were infected with the indicated strains for either 40 or 100 minutes and then stained with a membrane marker (CD11b) and analyzed by fluorescent microscopy. Individual bacteria were scored for being membrane bound or internalized by examining each individual Z-plane image and the percentage of internalized was plotted. The data shown is representative of multiple experiments done with similar results in which >40 macrophages in each experiment were analyzed. (D) Peritoneal macrophages were infected with the indicated strains for either 40 or 100 minutes. Prior to each sample point, gentamicin was added to some of the wells to kill extracellular (and/or surface-exposed) bacteria. The percentage of gentamicin sensitive bacteria was derived by comparing the number of colony forming units (CFU) recovered from three independent wells per condition. Shown is a representative data set of a single experiment repeated several times with similar outcomes. (* *P* < 0.05 using student *t*-test)

There is evidence that the enteropathogenic *Yersiniae* exports their type III effectors to the bacterial cell surface and that this secretion step is followed by a distinct process that translocates the surface-bound effectors across the host cell membrane [14–16]. The latter translocation step requires the pore-forming YopB protein: a *ΔyopB* mutant strain exports the type III effectors normally from the bacterium, but during infection is unable to translocate those effectors into the host cell [8]. To test whether YAC formation is dependent on YopB function, peritoneal macrophages were simultaneously labeled with pHrodo and infected with either YPIII/pYV or YPIII/pYV/*ΔyopB* bacteria. Following a 40-minute infection period, *ΔyopB* bacteria induced the formation of YACs at comparable levels as YopB-expressing bacteria (see Fig 6B). However, if macrophages were first infected for 60 minutes with these strains and then labeled with pHrodo for 40 minutes (similar to the experiment shown in Fig 4), whereas the majority (89%) of YPIII/pYV bacteria were able to maintain the acidified compartment (Fig 6A and 6B; *arrow 1*, pHrodo* positive; arrow 2, pHrodo* negative), there was a significant reduction (46.3%) of YPIII/pYV/*ΔyopB* bacteria in acidified compartments in identical infection conditions (*arrow 3*, pHrodo* positive; arrow 4, pHrodo* negative). These data suggest that while the formation of YACs is not dependent on type III effectors gaining access to the macrophage cytosol, the relative stabilization of this niche requires effector translocation across the macrophage plasma membrane.

To the best of our knowledge, a YopB-independent activity has so far not been described for type III effectors in *Y*. *pseudotuberculosis* or other Gram-negative species in which YopB-like proteins mediate effector translocation into the host cell cytosol. As mentioned above, one unambiguous activity of *Yersinia*’s type III secretion system is to resist phagocytosis (see Fig 1 and [4]). It was therefore tested whether YopB contributes to *Yersinia*’s anti-phagocytosis activity by comparing phagocytic activity of macrophages infected with YPIII/pYV, YPIII/pYV/*ΔyopB*, or YPIII/ΔpYV bacteria. Peritoneal macrophages were infected with these strains for either 40 or 100 minutes and then stained with a plasma membrane marker to distinguish between surface-bound and internalized bacteria. After 40 minutes of infection 8% YPIII/pYV and 27% YPIII/pYV/*ΔyopB* bacteria were internalized which significantly differed from that of YPIII/ΔpYV bacteria in which 59% were internalized (Fig 6C). After 100 minutes of infection the level of internalized YPIII/pYV bacteria remained unchanged (8%). In contrast, at this later time point the level of internalization of YPIII/pYV/*ΔyopB* bacteria did not significantly differ from that observed for YPIII/ΔpYV bacteria (62% and 58%, respectively). The relative anti-phagocytic activities of these strains were also assessed by treating infected cultures with the antibiotic gentamicin which kills surface-bound bacteria but not internalized bacteria due to the membrane impermeability of gentamicin. Only bacteria which are in direct contact with the extracellular medium will be killed by the gentamicin. Following a brief infection period (40 minutes), the percentage of gentamicin sensitive, macrophage-associated YPIII/pYV and YPIII/pYV/*ΔyopB* bacteria were both approximately 90% (Fig 6D). After prolonged infection (100 minutes), the percentage of gentamicin sensitive YPIII/pYV remained unchanged (90%) whereas the gentamicin sensitivity of YPIII/pYV/*ΔyopB* bacteria was substantially reduced (20%). These data are consistent with the microscopic-based results further supporting that there is a transitory YopB-independent anti-phagocytic activity of the *Yersinia* type III secretion system (or possibly other factors encoded on the pYV virulence factor, e.g., the attachment factor YadA). These data also show that the majority of membrane embedded YPIII/pYV bacteria are sensitive to gentamicin. Consistent with the data shown earlier in which pHrodo could diffuse into *Yersinia*-containing compartments in an on-going infection (see Fig 4), the sensitivity of YPIII/pYV *Yersinia* to gentamicin can only be attributed to the bacteria being located inside the host-membrane bound compartments that are exposed to the extracellular medium. Initially, YACs formed by YPIII/pYV/*ΔyopB* bacteria are fluid accessible (while they are gentamicin sensitive) but, eventually get internalized into the host cell thereby becoming resistant to the antibiotic.

### The acidic component of YACs enhance *Yersinia* survival

**Fig 7:**
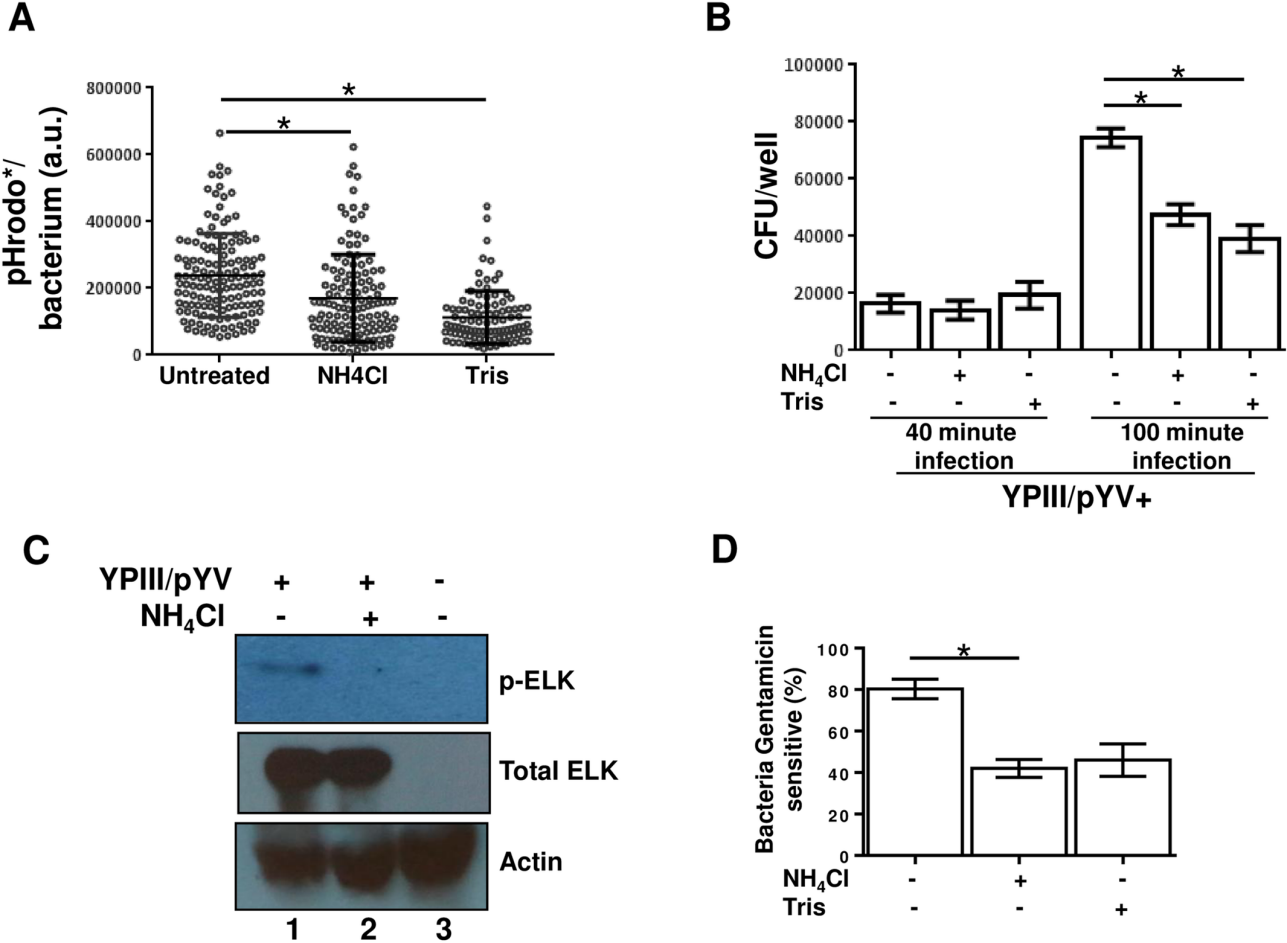
Acidification of YACs contributes to the survival of macrophage-associated *Yersinia*. (A) RAW macrophages were either left untreated or separately treated with NH_4_Cl or Tris (pH 7.4) 60 minutes before being infected with YPIII/pYV. After 60 minutes of infection, macrophages were labeled with pHrodo and infected a further 40 minutes and the fluorescence of individual macrophage-associated bacteria (N >100 each condition) is plotted. Shown is a representative data set of a single experiment repeated several times. (B) Peritoneal macrophages were either left untreated or separately treated with NH_4_Cl or Tris 60 minutes prior to being infected with wild-type *Yersinia* (YPIII/pYV) for 40 or 100 minutes. Infected macrophages were then washed and lysed and the viable bacteria were enumerated by CFU assay. Shown is a representative data set derived from three independent wells per condition of an individual experiment that was repeated multiple times. (C) Peritoneal macrophages were either left untreated or treated with NH_4_Cl for 60 minutes and then infected with *Y*. *pseudotuberculosis* expressing a *yopE*_*1–120*_ *–ELK* fusion construct for 90 minutes. Macrophages were then lysed in sample buffer and the levels of phosphorylated YopE_1–120_-ELK, total YopE_1–120_-ELK, and actin were determined by western blotting. YopE_1–120_-ELK exclusively becomes phosphorylated by host cell kinases following its type III-mediated translocation into macrophages. This experiment was repeated with similar results three times. (D) Peritoneal macrophages were treated with NH_4_Cl or Tris as described and infected with YPIII/pYV for 100 minutes. Prior to terminating the infection gentamicin was added to some of the wells as described for Fig 5D. Shown is a representative data set derived from three independent wells per condition of a single experiment repeated several times with similar outcomes. (* *P* < 0.05 using student *t*-test)

Macrophages were treated with either of two different pH-raising agents to determine whether the relatively acidic environment of YACs impacts infection. Treating macrophages with either membrane-permeable ammonium chloride (NH_4_Cl) or membrane-impermeable Tris (pH 7.4) greatly reduced the pHrodo* acid-activated fluorescence that is normally generated following infection of macrophages by YPIII/pYV bacteria (Fig 7A and S2 Fig). Other than loss of the acid-dependent pHrodo* fluorescence, treatment of macrophages with either NH_4_Cl or Tris did not notably affect the infection process, based on microscopic examination, after 40 minutes of co-incubation. However, at longer infection times (100 minutes), significantly more YPIII/pYV bacteria were physically associated with treated compared to untreated macrophages (S3 Fig). To assess the viability of these macrophage-associated YPIII/pYV bacteria, macrophages were osmotically lysed following 40 or 100 minutes of infection and the number of live bacteria was determined by a colony forming unit (CFU) assay. Untreated and NH_4_Cl- or Tris-treated macrophages yielded comparable CFUs following 40 minutes of infection. After 100 minutes of infection, the number of CFUs recovered from treated macrophages was notably less than that recovered from untreated macrophages (Fig 7B). Hence, even though the number of bacteria associated with the macrophage is greater in NH_4_Cl- or Tris-treated conditions, the viability of these bacteria is markedly reduced. When cultivated in the absence of macrophages, the proliferation of YPIII/pYV bacteria was not detectably affected when incubated in NH_4_Cl—or Tris-supplemented media (S4 Fig). To determine whether the formation of YACs were dependent on host cells proton pumps, macrophages were treated with bafilomycin prior to and during infection. Whereas the overall morphology of cells treated with bafilomycin were greatly affected, treatment did not visibly affect the pHrodo* signal generated by YPIII/pYV (*data not shown*). Taken together, these data suggest that the reduction in pH is a bacterially-driven process that serves to promote the survival of the pathogen.

The reduced viability of YPIII/pYV bacteria in neutralizing conditions occurring between 40 and 100 minutes of infection coincides with the YopB-dependent stability of YACs as well as the YopB-dependent resistance to phagocytosis. Since YopB-dependent translocation plays a critical role in the survival of macrophage-associated *Y*. *pseudotuberculosis* [17], it was tested whether neutralizing conditions impact the translocation of type III effectors into the macrophage cytosol. Macrophages were infected with a YPIII/pYV strain expressing a YopE_1–120_-ELK hybrid protein that is secreted by the type III secretion system. Following its translocation into the host cell cytosol, the ELK epitope of YopE_1–120_-ELK becomes phosphorylated by host cell kinases providing readout for type III-mediated translocation [11]. Phosphorylated YopE_1–120_-ELK was readily detected in untreated macrophages infected with YopE_1–120_-ELK-expressing YPIII/pYV (Fig 7C). In contrast, there was no detectable phosphorylation of YopE_1–120_-ELK following a comparable infection of ammonium chloride-treated macrophages. Reduced type III-mediated translocation of effectors in neutralizing conditions suggests that optimal functioning of this virulence-associated organelle occurs in a low pH environment. As mentioned earlier, one notable function of the type III section system is inhibiting phagocytosis. There was an enhanced level of phagocytosis in NH_4_Cl- or Tris-treated macrophages as measured by gentamicin protection (Fig 7D) consistent with a reduction in type III activity in neutralizing conditions. Collectively these findings indicate that *Yersinia*-driven acidification of YACs promotes the optimal functioning of its type III secretion system.

### The type III effector YopJ enhances YAC stability

**Fig 8:**
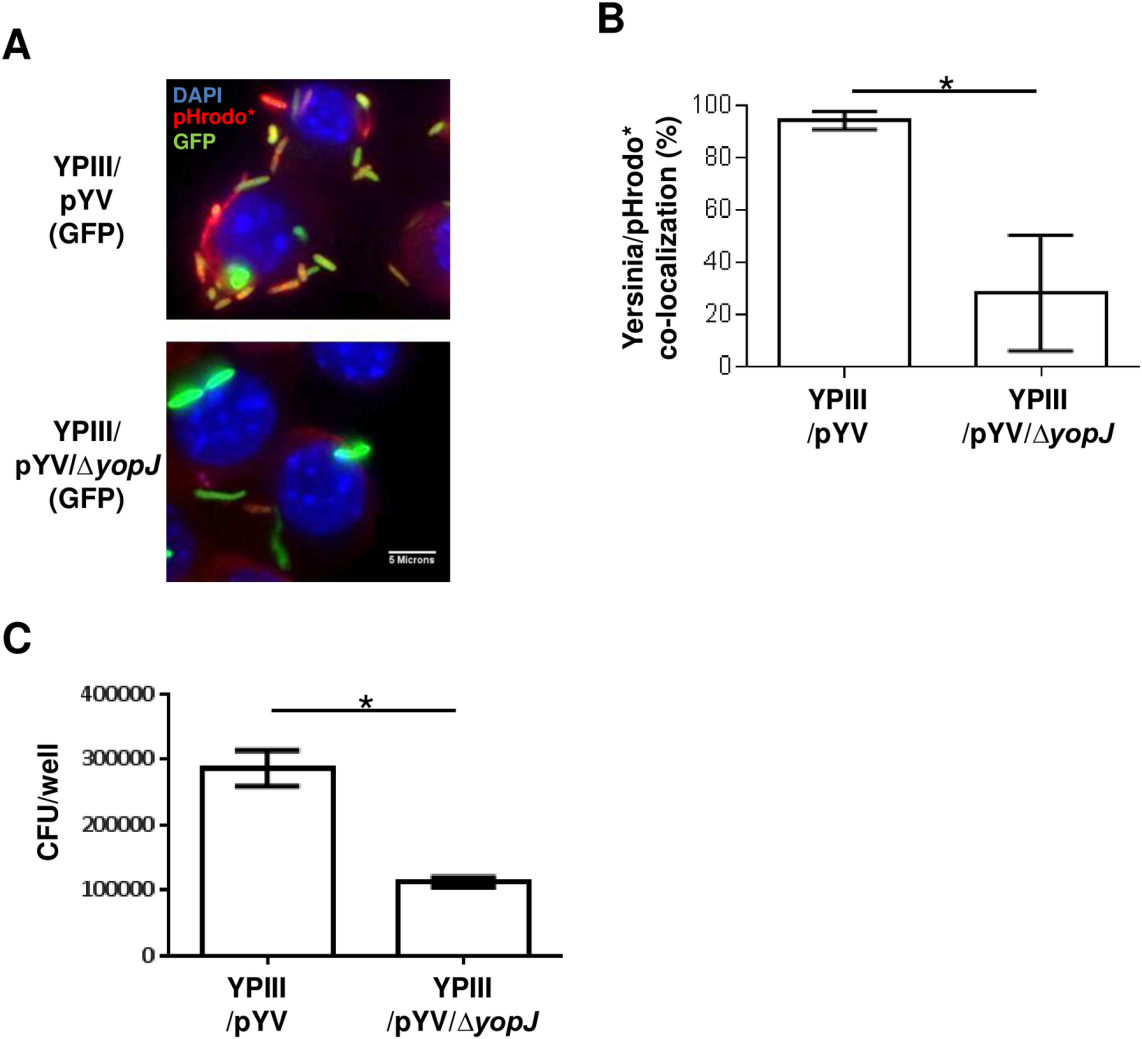
The type III effector YopJ stabilizes YACs. (A) RAW macrophages were infected with GFP-expressing wild-type (YPIII/pYV) or YopJ-deleted mutant (YPIII/pYV/Δ*yopJ*) strain for 60 minutes and then labeled with pHrodo for 40 minutes as described for Fig 4. (B) The percentage of bacterial cells co-localized with pHrodo was generated by counting bacteria associated with >40 infected cells in multiple independent experiments. (C) Three independent wells of RAW macrophages were infected with the indicated strain for 90 minutes. The number of macrophage-associated viable bacteria were then determined as described in Fig 6B. Shown is a representative data set of a single experiment repeated several times. (* *P* < 0.05 using student *t*-test)

The association between the type III secretion system and YACs was further characterized by infecting macrophages with several *Y*. *pseudotuberculosis* mutant strains, each deleted for a different type III effector. Of the mutant strains tested, there was an evident difference in macrophages infected with a YopJ-deleted strain (YPIII/pYV/*ΔyopJ*) compared to macrophages infected with the isogenic YopJ-expressing strain YPIII/pYV. After 90 minutes of infection, the percentage of YPIII/pYV and YPIII/pYV/*ΔyopJ* bacteria inside acidified compartments was 90% and 28%, respectively (Fig 8A and 8B). This reduction in acid-activated pHrodo* fluorescence in macrophages infected with YPIII/ pYV/*ΔyopJ* was accompanied with a reduction in the number of macrophage-associated viable bacteria (Fig 8C). The YPIII/pYV/*ΔyopBΔyopJ* double mutant exhibited the same phenotype as the YPIII/pYV/*ΔyopB* strain (*data not shown*) suggesting that this activity of YopJ, like that of its other known cellular activities, requires it to be translocated into the host cell by a YopB-dependent process. Collectively, these data further support the linkage of acidic conditions and *Y*. *pseudotuberculosis* survival along the bacterium-macrophage boundary and indicates that YopJ contributes to the stabilization of these compartments.

## Discussion

The enteric yersiniae (*Yersinia pseudotuberculosis* and *Y*. *enterocolitica*) employ two very different cellular infection behaviors during their interaction with the mammalian host. In the initial phase of the infection these pathogens actively invade cells lining the intestinal tract. This ‘pro-invasion’ phase of the host infection cycle is dependent on chromosomally-encoded factors. Following their passaging across this host barrier and upon interacting with innate immune cells, these pathogens switch to a non-invasive mode in which they actively inhibit phagocytosis. This ‘anti-invasion’ phase of the host infection cycle is largely dependent on factors encoded on the ~72-kb virulence plasmid. For both infection modes, there have been relatively few studies on the cellular architecture along the *Yersinia*-host cell boundary. As noted earlier, Sarantis and colleagues [3] recently showed in a ‘pro-invasion’ model of infection using epithelial-like cells and a *Y*. *pseudotuberculosis* strain lacking the virulence plasmid (equivalent to YPIII/ΔpYV used in this study), a structure they termed a ‘pre-vacuole’ formed at the cell-pathogen interaction zone. This bacterium-containing pre-vacuole was characterized as a membrane-bound compartment that is fluid-accessible but with a pore size that excluded antibodies and complement factors and that eventually becomes fully internalized. Here we showed both dynamically and architecturally that the interaction of virulence plasmid-possessing *Y*. *pseudotuberculosis* and phagocytic cells substantially differs from that described for plasmidless *Y*. *pseudotuberculosis* and epithelial cells.

It has long been recognized that *Y*. *pseudotuberculosis* employs its virulence plasmid-encoded type III secretion system (T3SS) to counteract host cell phagocytic mechanisms [4]. Although the majority of the bacteria are successful in resisting becoming phagocytized when the T3SS is fully engaged (as shown in Fig 1), a fraction *Y*. *pseudotuberculosis* (8–15%) are internalized by macrophages, and to some extent, are able to survive and replicate in non-acidified autophagic compartments [18–20]. Here we physically and chemically characterized the niche that supports the majority of *Y*. *pseudotuberculosis* that do resist phagocytosis and stably reside on the surface of the macrophage. Our data indicate that during its extended residency on the surface of the macrophage, *Y*. *pseudotuberculosis* becomes partially embedded within the plasma membrane. Remarkably however, these bacteria that are so intimately associated with the macrophage plasma membrane, are not internalized but remain in contact with the extracellular milieu as evidenced by direct visualization and their accessibility to exogenously added reagents (e.g., pHrodo, gentamicin, neutralizing agents). The fact that these compartments are devoid of early endosomal and lysosomal markers EEA1 and LAMP1 indicates that they are neither typical early endosomes, nor phagolysosomes, and thus indicates that these compartments are likely fundamentally differ from the pre-vacuole compartments described above that form when plasmidless *Y*. *pseudotuberculosis* infects epithelial cells.

An entirely unanticipated finding was that these *Yersinia*-containing compartments rapidly acidify and that this acidification appears to be key in maintaining the integrity of these compartments and eventual survival of the bacterium. The absence of both early and late endosomal markers from these *Yersinia* acidic compartments (YACs) suggest that they are not products of a truncated phagocytosis and that their acidification is not derived from fusion between lysosomal compartments and the plasma membrane. When also considered with the fact that acidification was not affected by inhibition of host cell proton pumps and insensitive to inhibitors of endosomal transport, could indicate that the bacteria themselves are the source of the acidic conditions. Since the regions of lowered pH occurred in the immediate vicinity of the bacteria, it may be that the acid-generating factors become trapped in the confined microenvironment between the bacterial cell and the plasma membrane as visualized in Fig 2A.

The acidification of these compartments is largely dependent on *Yersinia*’s pYV-encoded factors but, surprisingly, did not depend on the type III effectors being translocated into the host cell cytosol. To the best of our knowledge, this is the first report of a function associated with secreted (coating the bacteria) but not translocated (injected), type III effectors. This ability of the translocation-defective strain (YPIII/pYV/Δ*yopB*) to form acidic compartments correlated with it delaying phagocytosis similar to the translocation-competent strain (YPIII/pYV). However, whereas YPIII/pYV bacteria were able to maintain the acidic compartments with longer infection periods (>40 minutes), there was a substantial loss of acidity of YPIII/pYV/Δ*yopB*-containing compartments; this loss of acidity is temporally linked to increased phagocytosis (see below). These findings suggest that translocation of type III effectors into the infected cell cytosol (mediated by YopB) is important for extending the longevity of these YAC compartments.

YopJ may be the ‘YAC longevity’ factor based on the fact that YPIII/pYV/Δ*yopJ* bacteria were defective in maintaining the low pH environment. This is a newly described activity of YopJ which may be linked to its previously described functions of inhibiting stress-induced signaling in cell-based models and inflammatory responses in mouse-based infection models [21,22]. For example, we have recently shown that in *Y*. *pseudotuberculosis*, YopJ impairs the maturation of dendritic cells [5] and others have shown similar findings in the related pathogens *Y*. *pestis* and *Y*. *enterocolitica* [23,24]. The relatively normal maturation of dendritic cells infected with YPIII/pYV/Δ*yopJ* bacteria could conceivably be due to its enhanced internalization which is a key requirement for dendritic cell maturation. Thus it remains to be determined whether the acidic environment that is created by *Yersinia* is a cause or an effect of its various type III-dependent activities.

The loss of acidity in YPIII/pYV/Δ*yopB*- and YPIII/pYV/Δ*yopJ*-containing compartments, or through the exogenous treatment of neutralizing compounds to cells infected with YPIII/pYV, were correlated with increased internalization (i.e., loss of anti-phagocytosis) and reduced bacterial viability. Since neutralization of these compartments leads to their internalization, it implies that the loss of acidic conditions allows the endocytic pathway to re-engaged. These neutralization-dependent outcomes are likely due to it causing a reduction in the translocation of type III effector proteins into the cytosol of the macrophage. Previously Skrzypek and colleagues [25] previously showed that bafilomycin (which inhibits host protein pumps) did not impact the translocation of YopE into eukaryotic cells which, when considered with our own findings that this inhibitor did not affect YAC formation, further supports a model in which *Yersinia* is driving the acidification of these compartments to promote their survival. This situation may be similar to that of *Helicobacter pylori* which promotes its survival in the stomach by metabolizing urea resulting in a localized increase in pH [26].

As detailed by Edgren et al. [27], there are a number of observations indicating that the type III effectors of *Y*. *pseudotuberculosis* may be translocated into the host cell cytosol via a similar mechanism by which binary AB toxins enter cells. A particularly clear example of how pH can drive AB toxin membrane translocation is that of diphtheria toxin [28]. Recently it has been shown that the acidic pH of the endosomal lumen causes a partial unfolding of this toxin exposing hydrophobic surfaces that in turn promotes membrane insertion and subsequent translocation of the toxin [29]. If a similar pH-dependent mechanism is driving the translocation of T3SS effectors, the findings presented here would indicate that during its initial interaction with the macrophage *Y*. *pseudotuberculosis* must rapidly create a suitable translocation-friendly local environment that is disengaged from the endosomal maturation process.

## Supporting Information

S1 Fig Relationship between pHrodo*-derived fluorescence and level of infection in individual cells. The total cellular fluorescence generated in Fig 2B was plotted as a function of the number of bacteria associated per infected macrophage. The linear regression was calculated and the resulting *r* ^2^ values for the YPIII/pYV plot is 0.375 and for the YPIII/ΔpYV plot is 0.020. (* *P* < 0.05 using student *t*-test)
(TIF)

S2 Fig Treating macrophages with NH_4_Cl or Tris (pH 7.4) reduces the acidity of YACs. Macrophages were either left untreated or treated and infected as described in Fig 4. The plot generated from the quantification of the pHrodo* signal is shown in Fig 4A.
(TIF)

S3 Fig Treating macrophages with NH_4_Cl or Tris (pH 7.4) impacts the infection dynamics between *Yersinia* and macrophages. Macrophages were either left untreated or treated and infected as described in Fig 4. Following infection, macrophages were stained for membrane (CD11b) and the number of *Yersinia* per macrophage was enumerated and plotted.
(TIF)

S4 Fig Neither NH_4_Cl nor Tris affects growth of *Yersinia* in the absence of macrophages. Wild-type *Yersinia* (YPIII/pYV) was grown overnight at 27°C and then diluted to an optical density (OD) of 0.05 in tissue culture media and grown for 2 hours. The cultures were then switched from 27 to 37°C for one hour to induce the expression of genes encoding the type III secretion system. The cultures were diluted again to an OD of 0.05 in tissue culture media supplemented with either 30mM ammonium chloride (NH_4_Cl) or Tris base and grown for 2 more hours at 37°C in order to mimic the conditions used in infection experiments. The OD was recorded at T = 0 and at 2 hours. The data shown is representative of several experiments with similar results.
(TIF)
