# Supplementary figures and images for: Pathogenic Yersinia Promotes Its Survival by Creating an Acidic Fluid-Accessible Compartment on the Macrophage Surface
Source: PLoS One. 2015 Aug 14;10(8):e0133298. doi: 10.1371/journal.pone.0133298 (PMC4537277; doi:10.1371/journal.pone.0133298)

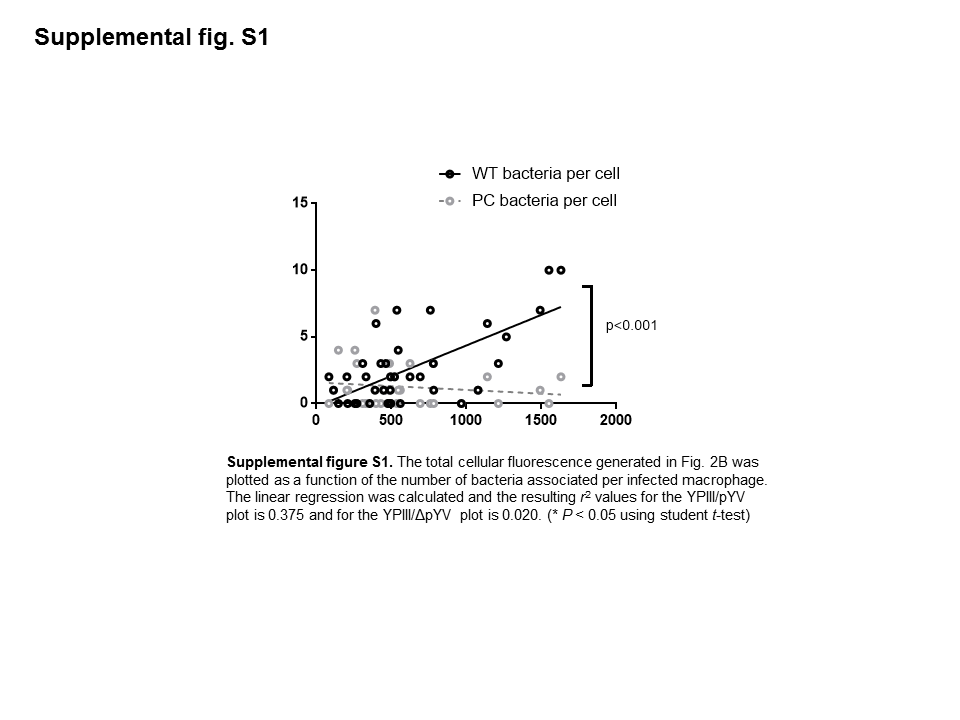

Supplement: S1 Fig — The total cellular fluorescence generated in Fig 2B was plotted as a function of the number of bacteria associated per infected macrophage. The linear regression was calculated and the resulting r 2 values for the YPIII/pYV plot is 0.375 and for the YPIII/ΔpYV plot is 0.020. (* P < 0.05 using student t-test) (TIF) [file pone.0133298.s001.TIF]

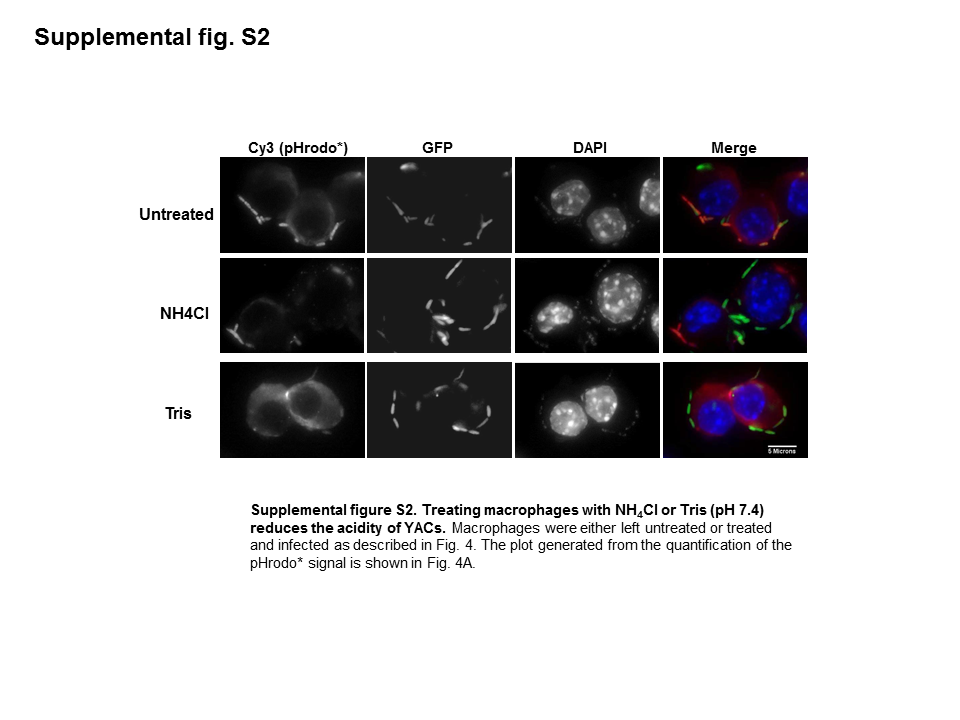

Supplement: S2 Fig — Macrophages were either left untreated or treated and infected as described in Fig 4. The plot generated from the quantification of the pHrodo* signal is shown in Fig 4A. (TIF) [file pone.0133298.s002.TIF]

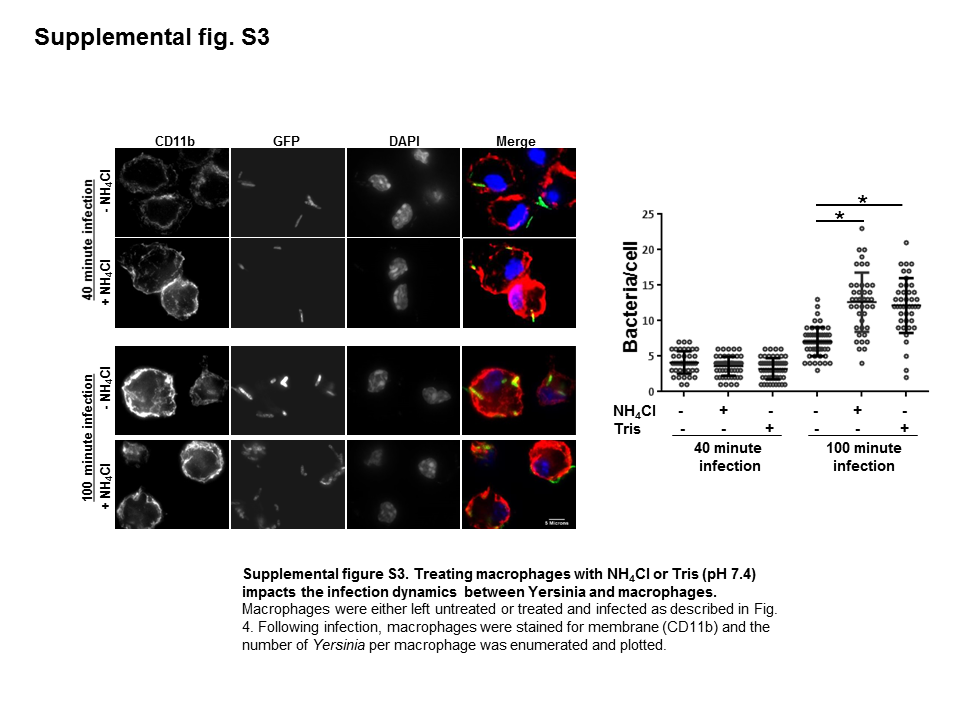

Supplement: S3 Fig — Macrophages were either left untreated or treated and infected as described in Fig 4. Following infection, macrophages were stained for membrane (CD11b) and the number of Yersinia per macrophage was enumerated and plotted. (TIF) [file pone.0133298.s003.TIF]

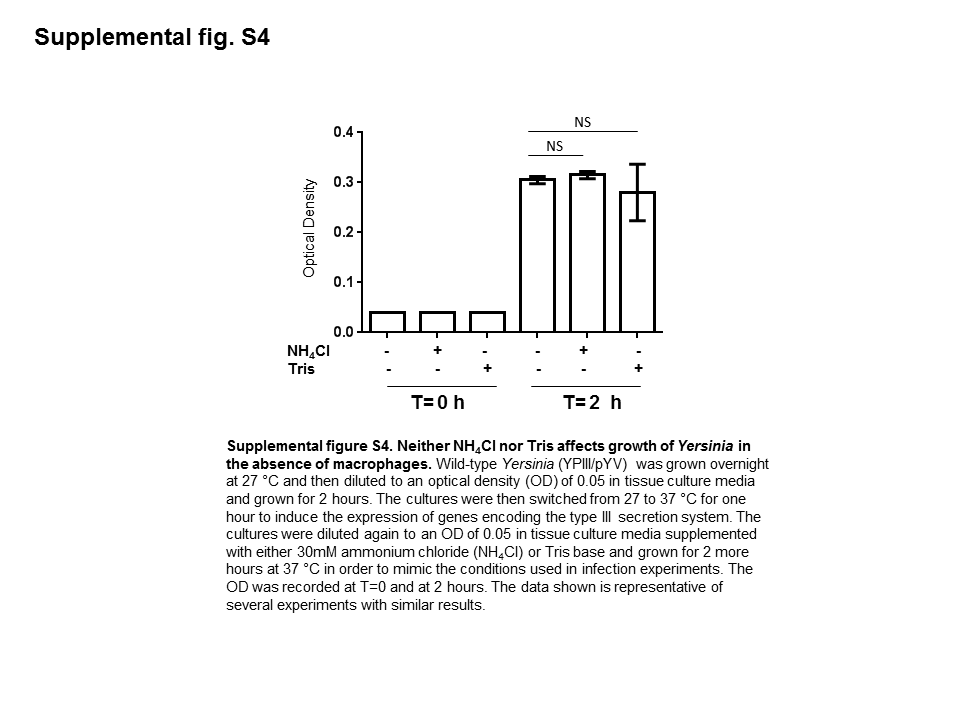

Supplement: S4 Fig — Wild-type Yersinia (YPIII/pYV) was grown overnight at 27°C and then diluted to an optical density (OD) of 0.05 in tissue culture media and grown for 2 hours. The cultures were then switched from 27 to 37°C for one hour to induce the expression of genes encoding the type III secretion system. The cultures were diluted again to an OD of 0.05 in tissue culture media supplemented with either 30mM ammonium chloride (NH4Cl) or Tris base and grown for 2 more hours at 37°C in order to mimic the conditions used in infection experiments. The OD was recorded at T = 0 and at 2 hours. The data shown is representative of several experiments with similar results. (TIF) [file pone.0133298.s004.TIF]
